# Precocious and Other Phenomena of Sexual Orgasm

**Published:** 1878-06

**Authors:** James Nevins Hyde

**Affiliations:** 117 S. Clark Street; Chicago


### Original Communications.

### PRECOCIOUS AND OTHER PHENOMENA OF SEXUAL ORGASM.

By James Nevins Hyde, Chicago.

In the light of recent observations, few will refuse to admit that sources of irritation in the external genitals, may induce reflex disorders of the most varied and exaggerated character. In a recently-published paper ([1]), I endeavored to describe some of the consequences of præputial stenosis, with its complications of retained smegma, etc., and briefly alluded to the results of stenosis of the urinary meatus.

In the present article, I desire to adduce some observations made by myself and others, which serve to illustrate the centric, rather than the excentric, origin of certain precocious and other unusual phenomena of sexual orgasm, in infancy and youth. Without question, it is far more easy to decide that these are the results of influences operating at the nervous centres, than to decide as to the nature of such influences. In one case, certainly, I think it will be seen that hyperæmia of the nervous ganglia at the base of the brain, was an important factor. In others, the same results may have been due to disturbance in the nutrition of these ganglia, or to obscure states of exalted irritability of the sensory portions of the brain, spinal cord and peripheral sensory nerves, or to changes in the vascularity of the cerebral or spinal meninges, or to changes in the quality of the circulating fluid itself. In two instances, a strong probability is suggested that

(1). Præputial Stenosis: its possible complications and consequences. *Ch. Med. Jour. & Ex.* Ap., 1877, p. 295.

the morbid conditions of the nervous system were the result of hereditary influence.

Without attempting to discuss this question, whose solution must depend upon the accumulation of many more facts and anatomico-pathological data, I proceed to the consideration of the phenomena themselves.

In the early part of April, 1878, Dr. A. B. Newkirk, of Hyde Park, Ill., described to me, in the course of a conversation, certain peculiar symptoms of an obscure character, which were displayed by two young patients in his professional charge. It occurred to me that his description was in many particulars identical with that given by Prof. A. Jacobi, of New York, in his admirable monograph, entitled, "On Masturbation and Hysteria in Young Children," (1) where the details are given of several cases, one of which in this connection has a special interest. I suggested to Dr. Newkirk that he procure and read the paper referred to ; and this he not only did, but became convinced, after further observation of his patients, that our suspicion as to the character of their disorder was not without foundation. He further kindly invited me to join him in making a careful examination of the children, and permitted me to publish the results.

The father of the little patients, whose cases are briefly described below, is 38 years old, slender in frame and of a nervous temperament. He has always enjoyed good health, never having suffered from any severe disease nor grave lesion. The mother, 31 years of age, is also of nervous temperament. She is slender in frame, but her cheeks exhibit the red hue of health. Since youth she has worn glasses to correct myopia, but, like her husband, she has never had any disorder originating in the nervous centers, nor other serious disease. They have been married eleven years, and the two children, named below, are the sole fruit of the union.

The eldest of these latter, R. J., æt. six years, was brought into the world after natural labor, enjoyed invariably good health

(1). Reprint from the *Amer. Jour. of Obstetrics and Diseases of Women and Children*, Vol. VIII., No. 4, Feb., and Vol. IX., No. 11, June. 1876.

up to the age of six months, and, with the single exception of what was called "scarlet rash," has had none of the diseases of infancy. At this time, his mother first noticed the occurence of the peculiar symptoms since exhibited. The baby, whether seated in its crib, on the floor, in its high chair, or even when placed upon its hands and knees, would suddenly become rigid. His expression would be that of one "abstracted,"—his gaze fixed. The hands, in which his toys happened to be held (on the first occasion, when observed, a clothes-pin was grasped), were firmly and forcibly closed upon the objects which they contained, to such an extent that they could be removed only with considerable difficulty. The body and limbs also became rigid, and the thighs crossed, the one over the other. He would then "work himself" for a longer or shorter period of time, when a copious perspiration would occur, and the scene be ended. Usually this was succeeded either by sleep or by a complete return to the natural expression and behaviour of the infant. These phenomena were never observed during sleep; just before he slept, however, and immediately after awaking, they might generally be expected.

When the child was in this condition, the act was immediately discontinued if he were suddenly and rather sharply addressed, or if his attention were diverted by the approach of strangers, the offer of toys, etc., or if he were taken up in the arms. So soon, however, as the attention ceased to be thus diverted, the child usually relapsed into the same condition. As to the intervals between these attacks and their relative frequency, no very precise information could be obtained.

The mother hoped that she would notice some change in this particular as soon as the first dentition was completed, and indeed was encouraged in this hope by those whose advice she had sought. When the infant was eighteen months old, he was weaned; and then occurred the longest interval between the accesses which up to that time had been noted—a period of four weeks. Meantime the child had learned to walk. Soon, however, after the eruption of the eye and stomach teeth, the phenomena described above recurred, and have continued with various intermissions, worse in the summer than in the winter, up to the present time.

Latterly the boy displays these symptoms, in and out of doors, without regard to the presence of strangers, on the floor, on the ground, and, on several occasions, upon the broad top of the fence in front of the house. The only difference noted, between his condition at the time of these occurrences and that observed in infancy, is that his limbs are crossed in a more marked manner. On one occasion, when observed by Dr. Newkirk, he was on his hands and knees upon the floor, the trunk supported entirely by the hands, and the right leg thrown over the left so as to forcibly approximate the thighs: posteriorly the body was supported on the left knee. In this position, a certain degree of twisting or swaying motion was perceptible, followed by perspiration and sighing, when the acts were discontinued. At my request, the boy assumed the precise position described, when upon the floor: and also upon a low arm-chair, when his knee rested on the seat and his hands clutched the arms.

The only result noticed by the parents was a change in the child's temper. When aroused from the peculiar state indicated, he seemed cross and irritable, though at other times he possessed a cheerful and happy disposition. He had been rarely seen handling the penis — so rarely, indeed, that they were sure no such habit existed. There had never been dysuria nor nocturnal diuresis, to an extent greater than that noted in healthy children. Once or twice only, had he complained of "soreness" about the genitals, which always proved to be temporary in character. At one time the mother thought she noticed a tendency to stumble when walking, but this also had ceased to attract her attention.

During the last few years the habit had not to any marked extent either increased or decreased. At one time during the last summer, the father had taken the child with him out of doors for play, while the former was engaged in his usual occupations, and he then noticed that sometimes for an entire day the boy would seem to be engaged in the performance of the act. The mother stated that occasionally for months at a time, he would seem to her to be engaged in nothing else.

When examined, the lad was found to be a blue-eyed and light-haired child, with well-developed head, body and limbs, intelligent expression, red cheeks and vivacious demeanor. His teeth were normal, axes of eyes parallel, senses perfect, and his health without the slightest evidence of impairment. In stature, weight and nutrition, he was entirely normal. There were no evidences of genital irritation, the prepuce was permeable by the glans, free, not redundant nor pocketed. His urine also was normal and passed without straining. Dr. Newkirk agreed with me in declaring that we could discover no symptom of ill-health nor lesion, with the single exception of a slight tendency to genu valgum, which, under other circumstances and in another child, would certainly have escaped notice. His appetite was good, bowel-dejections normal and regular, sleep undisturbed. His mother complained only that he was "restless and nervous."

The second child, G. A. J., æt. 17 mos., had a history not unlike that of his brother. He was brought into the world by natural labor, had never suffered from infantile disease, and, until he arrived at the 9th month, had never displayed symptoms which aroused special attention. At that age, however, his mother noticed at times certain singular manifestations, which she thinks would not have especially interested her, if she had not observed something similar in the case of her first infant. When the baby was left alone in the crib, it would occasionally assume the fixed and abstracted gaze described above, and its little limbs become rigid, but the lower extremities, instead of being crossed so as to approximate the thighs, as in the case of the brother, would be firmly extended and separated. At the same time, the opened hands were pressed, palm downward, into each iliac fossa. After a few moments of accelerated respiration, and very little if any facial and bodily movement, copious diaphoresis would ensue, and the child would either sleep or resume its natural aspect and demeanor. Most frequently these acts would occur just before the infant was placed in its crib for the purpose of taking his daily nap, but, from first to last, the attacks have proved much less frequent, prolonged and intense than in the first case. However, during some weeks, accesses of the same character would occur once in each day.

The child occasionally wet its bed—not oftener, however, than other children of the same age; and did not strain when urinating.

When examined, it was found to be a blue-eyed and light-haired infant, very well nourished and developed, of nervo-lymphatic temperament, its cheeks somewhat less rosy than those of its brother. It was able to sit up and to propel itself on the floor in various methods, chiefly by dragging the body forward, when in the sitting posture, by alternate flexion and extension of the legs. Dentition had progressed favorably ; there was nothing abnormal about the genitals. The expression was intelligent, the axes of the eyes parallel, the senses unimpaired. In brief, the most careful examination failed to detect any evidence of perverted function, disease or deformity.

In order to exhibit to the reader the similarity between these histories and the observations of Dr. Jacobi, I append a brief abstract of the most noteworthy of the cases recorded by the latter :

A female child was healthy during nine months, after which teething commenced. At the fourteenth month, she had nine teeth, and the dentition was unaccompanied by pain or constitutional disturbance. At this age, slight indications of nervous trouble were noticed when the child was lying in its mother's lap. She suddenly became pale, had a peculiar dazed expression, and her attention could not be readily attracted. When raised up and moved, she immediately became natural in looks and action. This was repeated only a few times, when the attacks changed in character. In addition to the appearance of the countenance already described, there much muscular rigidity: the arms became quite stiff, and strongly resisted being flexed: the hands were clenched, and the little fists firmly pressed into the iliac regions on either side. At the same time the legs were strongly extended at right angles to the body, and there was a strong contraction of the abdominal muscles and a straining as if at stool. If the child were held against one's breast, she made strong pressure with the knees and up and down movements of the body. After a short period, a moment or two, the respirations were quickened to a rapid panting, and perspiration started freely from the head and stood in drops about the mouth. The attacks often terminated in sleep. There was at no time any spasmodic or convulsive movement, or unconsciousness or mental disturbance beyond an apparent abstraction. The attacks occurred irregularly, with intervals of some days, or many times a day for several days in succession, and sometimes for two or three hours with but slight intermission. They never came on during sleep, but usually when the child was sitting on the lap, and occasionally when on the bed or floor. If she were placed on the floor early in the attack, and amused with her playthings, it would frequently be broken up; if however, she were held till it was fully developed and then put down, she would lie upon her side and the attack would progress as described.

The symptoms described in another case, that of a girl three years old, were, redness of the face, slight twitching about the eyes, and an occasional deep sigh, the attacks occurring when the child was playing upon the floor or crouched upon a chair. The accesses never occurred in sleep, or when walking, playing or tossing about. She was apt also to cross the legs or closely join the thighs. After violent friction of the limbs, with hurried respiration and purple hue of the face, the act was completed with perspiration and sighing.

Similar symptoms were displayed by a little boy, who would seat himself on the floor, stare upwards and commence a kneading exercise by means of his two little fists directed against his privates, which his nurse thought "very cunning." In this case, however, there were symptoms of vesical catarrh and enuresis.

The following points are noteworthy: 1. The early age at which the orgastic phenomena were first observed in Dr. Newkirk's cases, earlier in fact than the ages recorded by Dr. Jacobi, and, so far as I am aware, the earliest on record. 2. The almost imperceptible effect of the long continued orgastic excitement upon the well-being of mind and body, in the cases here first reported. Without repetition, it may be briefly said on this point, that the children in every particular did not come short of a high standard for the average mental and physical attainments of other children of their own age. 3. The co-existence of two rare cases in one family. This would tend to excite a suspicion of hereditary influence. It has been seen that the parents were both of a nervous temperament and slender frame. They had had but two children during eleven years of married life. Upon this point, of course, an opinion can only be expressed with reserve. 4. The very remarkable correspondence between the phenomena in all the reported cases. They do not differ by as much as many other diseases of the same name in different individuals. A description of one would answer fairly well for the others.

It should be added that with the judicious administration of camphor bromide, and a pursuance of orders to break up every attack when possible, the children have been gradually improving under Dr. Newkirk's management.

How far attacks similar to those described, and long continued, might operate in laying a foundation for future disease, it is impossible to say. I am inclined to believe that the history of the later life of these patients, might throw some light upon the possible results of these early accesses. At the same time it is to be remembered that nature is wonderfully conservative, and never more so than in youth, when drafts may be made upon the nervous forces which would utterly prostrate the adult. I had recently under my charge a young woman fifteen years of age, who admitted that she had had intercourse with impunity sixteen times in twenty-four hours. She was suffering then from inflammation of the vulvo-vaginal gland. Another patient, a young man of twenty, assured me he had had intercourse twelve times between 3 and 6:30 p. m. of the same day. On the other hand, a gentleman, past the middle age of life, a widower and a grandfather, had several liaisons with women in his neighborhood. Soon afterward, he became an inmate of the Insane Asylum of California at Stockton, and the accomplished superintendent of that institution, Dr. G. A. Shurtleff, wrote me, under date of April 7, that the patient was affected with general paralysis of the insane — a terribly fatal disease, from which there is not one well-authenticated case of recovery on record. Very recently I had under charge, associated with Prof. E. L. Holmes, a gentleman of fifty, in good circumstances, whose sexual excesses had produced a large obstinate corneal ulcer, which threatened at one time to become perforating.

In the matter of diagnosis, I cannot think of any disease whose symptoms might be confounded with those described above, unless it be chorea, and especially the hysterical and rhythmical forms recently described by Prof. Charcot, of Paris. (Vide *Jour. de Médecine et de Chir. Prat.*, t. 49, c. 4, Abstract from *Le Progrès Médical.*) Yet the latter have a marked individuality of their own. In rhythmical chorea, the head, trunk and limbs of one side of the body are incessantly agitated in alternate movements of flexion and extension, uniform and identical, the head bending on the trunk and the trunk on the pelvis. This occurs with mathematical regularity, night and day (except during sleep), when the subject is reclining or walking by the aid of another's hand, when the attention is fixed to, or distracted from any special object. The choreic movements, instead of resembling those of salutation, may suggest the motion of the blacksmith at his anvil, or the swimmer in the sea.

I have not given the name masturbation to the precocious orgastic acts described above, simply for the reason that it does not seem sufficiently precise. Its etymology alone (*manus stupratio*) supplies the objection.

Allied to these instances are those of precocious puberty, menstruation and pregnancy. Cases are on record of menstruation at the 9th month, at one year, and at one year and a half; and of pregnancy in the 5th year (Mandelshof), in the 8th (Carus), in the 9th (Jaubert), and in the 10th (Sims). One such has recently been reported by Molitor of Oberspallen (*Bulletin de l'Acad. de Médecine de Belg.*) The child menstruated in her 4th year, and, in the 8th, aborted with a hydatid mole and embryo, after intercourse with her cousin, 32 years of age.

It is difficult to see why intra-uterine perturbations of the nervous ganglia of the fœtus, which may produce congenital talipes and unilateral skin lesions of tracts supplied by a single nerve or series of nerves, should not at times be capable of producing premature puberal epochs.

In 1876, Dr. G. H. Nuckols, of Kentucky,(1) reported the case of a male infant, born Dec. 31, 1875, of a mother who contracted

(1) This report first appeared in the *Louisville Medical News*, June 10, 1876, p. 295. I incorporated a brief abstract of the case in the Digest of Literature of Hereditary Syphillis, prepared for the *Archives of Dermatology*, Jan., 1877, vol. iii, No. 2. Prof. Auspitz, of Vienna, reproduced my abstract in the *Vierteljahrschft. f. Derm. u. Syph.*, J. iv, Hft. 3. p. 423, and added that "priapism would hardly accord with the existence of hereditary syphilis." Since then, however, Th. Barsyphilis four years previously, the father having never suffered from the disease. On January 2d, the penis of the infant was found to be erect and as hard as cartilage; it remained in this condition thirty days. Symptoms of hereditary syphilis were displayed in the tenth week. Rapid improvement occurred under specific treatment.

I was recently consulted by a young man from Indiana, J. M., of sanguine temperament, for the relief of nocturnal emissions. He was well developed in head, trunk and extremities, and had a particularly frank and engaging expression of countenance. The genitals were normal in size and configuration; præputial opening, ample; urinary meatus, admitting sound No. 25 of the French scale; urethra, not hypersensitive when traversed by the same instrument. He had been subjected to a triple process of starving, sweating and freezing in a neighboring "water cure," and I at once placed him upon a generous diet with roborant medication, till he greatly improved in health, and his cheeks became rosy. I could discern no special indication for treatment of the genitals, until he informed me one day that his losses at night were always produced by masturbation in sleep. He would be aroused immediately after each act, partially sensible of what he had been doing, and with unmistakable evidences about him of his mishap. He had never practiced masturbation when awake, had never used tobacco in any form, and although at one time he had indulged rather freely in liquor, the habit had been abandoned before his present troubles commenced. At the time of making this explanation, he in fact wore the red ribbon of the temperance agitators. When asked why he had not earlier explained the precise character of his trouble in answer to the rather minute inquiries put to him, he replied that he thought that the nocturnal pollutions of all men occurred in the same manner. As usual in such cases, these accidents occurred at irregular intervals—sometimes several times in the night, somelow, of England, has found extensive nervous lesions in congenital syphilis, including new growths and gummata of nerves, atrophy of nerve cylinders, diminished lumen of cerebral capilaries, adhesions of dura-mater and arachnoid, exudation of greenish lymph, etc., changes capable of producing paretic priapism. I accept the great weight of Prof. Auspitz' authority in these questions, but believe that his statement, given above, requires qualification.

times on several successive days; occasionally with intervals of a week or two, rarely more.

On the 9th of March, 1878, after the production of anæsthesia, I perforated each side of the upper limb of his prepuce with a large triangular perineum needle and inserted a ring made especially for the purpose, of pure silver, leaving it *in situ.* This is very nearly the operation of infibulation, originally advocated and practiced by Celsus and his contemporaries, and still resorted to, I understand, by the superintendents of insane asylums in the treatment of the satyriasis of their patients. In this instance the ring was made with a peculiar lock, so that it could not only be fastened after its insertion but removed at pleasure afterward.

The patient returned to his home in Indiana and complained bitterly, in several letters, of disturbance at night and want of sleep in consequence of painful erections induced by the presence of the ring. I permitted him to remove it in fifteen days, when these troubles naturally ceased. On the 4th of May, he wrote that his habit had been broken up, and that he was, in accordance with my suggestions, contemplating matrimony.

A patient whose case presented some exceptional features, consulted me in June of 1877. He was 19 years of age, bilio-lymphatic temperament, and well nourished, his muscular system presenting an unusual development. The expression of his face was peculiar and not that of the average youth who masturbates, the peculiarity being due to an unusual prominence of the eyeballs. In consequence of this, the sclerotica was exposed in a wide ring about the cornea, highly suggestive of exophthalmos. He seemed quite intelligent and expressed the greatest grief over his condition.

He stated that when sixteen years of age he had, for a period of several months, made vain attempts at intercourse with a girl of his own age, whom he met in a clandestine manner almost every day. A necessary separation of the two occurred, and thereafter nothing unusual occurred, until in a few months he commenced to practice masturbation. These acts were repeated each morning, immediately upon his rising from bed, and were, as he stated, entirely beyond his control. They were preceded by a severe pain in the head, usually, but not invariably occipital, and then he seemed to lose control of himself. Without the slightest degree of sexual or erotic desire, he at once proceeded to perform the act with violent and even furious manipulations. At first seminal discharges were induced, but these, in his brutal performances, were soon succeeded by blood, and thereafter, nothing but the discharge of blood relieved him. On these occasions he experienced not the least pleasurable sensation but on the contrary severe pain. His tongue was coated, his bowels constipated, his sleep disturbed, his urine acid and loaded with urates, pulse and temperature normal. There was slight seborrhæa oleosa of the surface of the skin.

His external genitals had not the relaxed and shrivelled appearance which is too often observed in the confirmed masturbator. The testes were not shrunken but firm and full, the prepuce, wide, not redundant, and worn back of the corona glandis. The meatus admitted readily a No. 23 sound (Fr.). Its removal after insertion was followed by blood, which escaped from the urethra, congested in consequence of the severe mechanical irritation.

The brother of this patient was taken into our confidence, and every morning afterward for a month, he roused the patient early from sleep, douched the head, neck and chest in an abundance of cold water, and after a generous friction of the surface with a coarse towel, superintended the patient's dressing and advent at the breakfast table. This, with the free use of the alkaline bicarbonates (I prefer the salt of potass), regular evacuation of the bowels under the influence of cathartics, and the occasional employment of the bromides of potassium and sodium, resulted in relief. Two months afterward, the patient presented himself in a greatly improved condition both of mind and body. He had had but one access since the inception of his treatment, and this had been broken up by his brother. He has not consulted me since, and I am led to think the success was permanent, as he seemed fully sensible of his improvement and grateful for the relief afforded.

I conclude with a brief abstract of a case which seemed to me, when I first read a report of it in 1876, strikingly illustrative of the influence of centric disturbances in the production of onanism. The observation was made by Dr. Prevost, of Cambremer. (*L'Année Médicale*, *Caen*, No. 1, Vol II., p. 7, Dec. 1876):

X——, 22 years old, had an agreeable expression of countenance, brown hair, forehead slightly retreating, and was rather fully developed in the occipital region. He had left inguinal hernia, no prior interference with general health, and, though disposed to be isolated and taciturn, had a good reputation among his school-fellows. His parents were in robust health.

On Jan. 5, 1875, about 6 p. m., after a generous dinner, he retired to his room and locked the door. His mother, anxious in consequence of his behavior when at the table, followed, and through the keyhole saw him erect and fully dressed, engaged in the act of violent masturbation. This completed, he threw himself on his bed in his clothes and slept. The mother informed the father of what she had seen, and thereafter the young man was closely watched.

Nine days afterward, the patient left his friends at a picnic party in the woods, and this time the father followed him and witnessed the same scene as before. After returning home in the evening, the parent sternly reprimanded his son for his misconduct, when the latter informed him that he was very miserable, that for more than a year he had been subject to attacks of a furious sort in which masturbation became an irresistible necessity. He begged his father's forgiveness and promised that when he next had premonitions of his trouble, he would inform his friends, who might then secure his hands behind his back.

After dining on the 28th, he notified his father that he was about to be affected as before, and would soon be almost unconscious of what he was doing. His hands were immediately bound firmly behind his back, when he was at once seized with a convulsion that lasted for ten minutes. He fell to the ground, his respirations became accelerated, his face pallid. He also uttered hoarse cries in a strange voice. His father, thoroughly alarmed, hastened to liberate his son's hands, when the latter at once arose and in the presence of both his parents proceeded to perform the act of masturbation in the most furious manner, without pausing an instant. This over, he burst into tears, and concluded by falling asleep as usual. Dr. Prevost, who was then summoned, ascertained that the young man had no sexual desires. The first intimation he would have of the access, would be an insupportable pain in the back part of the head, occurring sometimes an hour or two before, sometimes immediately after meals; then there would be an erection of the penis and unconsciousness of subsequent events, so that the presence of strangers presented no bar to the execution of the act. On one occasion of this sort, when observed by his physician, the latter describes his condition as disgusting in the extreme, his face pale, his features contorted, saliva escaping from his mouth—the very picture of a satyr.

Under treatment of various kinds, hygienic, medicinal and dietetic, attacks did not fail to recur with tolerable regularity, February 4th, 12th, 21st; March 1st, 7th, 16th, 23d, and at nearly equal intervals up to July 2d. At this time leeches were ordered to be applied to the nucha as soon as the pain was perceived. On July 11th, an access of intolerable pain was felt, and fifteen leeches applied to the back of the neck. The access was aborted. On August 1, recurrence of pain, re-application of leeches, no attack. This proved to be the last. Eighteen months after, the patient had had no return whatever of his former symptoms.

117 S. Clark Street.

